# Inverse correlation between salt tolerance and host-adaptation in mycobacteria

**DOI:** 10.1186/s13104-016-2054-y

**Published:** 2016-04-29

**Authors:** Shady Asmar, Mohamed Sassi, Michael Phelippeau, Michel Drancourt

**Affiliations:** Unité de Recherche sur les Maladies Infectieuses et Tropicales Emergentes, CNRS, UMR 7278, IRD 198, Faculté de Médecine, Aix-Marseille Université, 27, Boulevard Jean Moulin, 13385 Marseille Cedex 5, France; Université de Rennes 1, Rennes, France

**Keywords:** Mycobacterium, Salt, Culture, *Mycobacterium tuberculosis*, *Mycobacterium canettii*

## Abstract

**Background:**

The genus *Mycobacterium* includes host-adapted organisms regarded as obligate and opportunistic pathogens and environmental organisms. Factors contributing to this wide range of adaptations are poorly known.

**Results:**

We studied the salt tolerance of 46 *Mycobacterium* species of medical interest. Representative strains of the *Mycobacterium tuberculosis* complex, *Mycobacterium avium* complex, *Mycobacterium chelonae*-*abscessus* complex, *Mycobacterium ulcerans*, *Mycobacterium marinum*, *Mycobacterium lentiflavum*, *Mycobacterium fortuitum* and *Mycobacterium conceptionense* were inoculated on Middlebrook 7H10 medium supplemented with 0–10 % sodium chloride. Colonies were counted after 2–4 week incubation at the appropriate 30–37 °C temperature depending on the tested strain. Further comparative genomics was done on 15 *Mycobacterium* strains representing the spectrum of salt-tolerance of mycobacteria. Based on the results the different species were grouped according to their salt tolerance into a “salt-sensitive” group (growth up to ≤3 % salt) containing the *M. tuberculosis* complex, *Mycobacterium chelonae*, *Mycobacterium lentiflavum, Mycobacterium ulcerans* and *Mycobacterium marinum*; a “salt-intermediate” group (growth between 4 and 6 % salt) comprising *Mycobacterium avium*, *Mycobacterium intracellulare*, *Mycobacterium chimaera* and a “salt-resistant” group (growth up to >6 %) comprising *Mycobacterium homonissuis*, *Mycobacterium bolettii*, *Mycobacterium fortuitum* and *Mycobacterium conceptionense*. Genomic analysis revealed that 290 genes were unique to species belonging to the salt-sensitive group; and that 15 % were annotated as being functionally associated with the ESX secretion systems Pro-Glu and Pro–Pro-Glu family proteins.

**Conclusions:**

In this work we found an inverse correlation between salt tolerance and host adaptation. We thus propose that salinity is one of the multiple factors determining the ecological niches of mycobacteria.

## Background

The genus *Mycobacterium* comprises more than 150 species [1]. The vast majority of these mycobacteria are environmental organisms found in soil and aquatic environments, with a few exhibiting some degree of host-adaptation, illustrated by their intra-amoebal survival [2] and variable pathogenicity in mammals and humans, culminating in well-adapted *Mycobacterium leprae* responsible for animal and human leprosy [3, 4] and *Mycobacterium tuberculosis,* complex organisms responsible for animal and human tuberculosis [5]. Later organisms exhibit the widest spectrum of niches from soil [6, 7], to amoeba [2] and mammals including humans [5]. The factors contributing to the survival of mycobacteria in one particular ecological niche are not yet fully understood.

Here, we explored salinity as one of the factors which could potentially affect the survival of mycobacteria in their ecological niches. More precisely, we focused our study on a few species of veterinary and medical interest, as they exhibit the broadest spectrum of ecological styles, from inanimate environments to amoeba and hosts.

## Methods

### *Mycobacterium* strains

A total of 46 *Mycobacterium* spp. strains were used in this study. They included 17 *M. tuberculosis* complex (MTC) isolates, including *M. tuberculosis* H37Rv CIP104475^T^ reference strain, 12 *M. tuberculosis* clinical isolates including two Beijing lineage isolates, *Mycobacterium bovis* Bacillus Calmette and Guérin (BCG), “*Mycobacterium canettii*” CIP 1400159 and two “*M. canettii*” clinical isolates (kindly provided by Prof. Eric Garnotel, Hôpital Laveran, Marseille, France); nine *Mycobacterium avium* complex (MAC) clinical isolates, including three *M. avium* clinical isolates, two *M. avium* subsp*. hominissuis* (here referred as *M. hominissuis*) clinical isolates, three *Mycobacterium intracellulare* clinical isolates and one *Mycobacterium chimaera* isolate; 11 *Mycobacterium chelonae*-*abscessus* complex clinical isolates, including three *M. chelonae* isolates, four *M. abscessus* isolates, three *Mycobacterium boletti* isolates and one *Mycobacterium massilliense* isolate; two *M. ulcerans* (ATCC19423 and ATCC33728); one *M. marinum*, three *Mycobacterium lentiflavum*, two *Mycobacterium fortuitum* and one *Mycobacterium conceptionense* clinical isolates. All clinical isolates were identified by 16S rRNA and *rpo*B gene PCR-sequencing as previously described [8]. *M. tuberculosis*, “*M. canettii*” and *M. ulcerans* strains were handled in a biosafety class 3 laboratory, while other mycobacteria were handled in a biosafety class 2 laboratory. Except for “*M. canettii*”, all clinical strains have been isolated and cultured by the authors. No ethical approval was required for this study. All mycobacteria were subcultured on Löwenstein-Jensen medium; (bioMérieux, Craponne, France), suspended in sterile phosphate buffered saline (PBS) and vortexed to complete homogenization of the suspension. As for clumping *M. tuberculosis*, *M. bovis* BCG, *M. abscessus* and *M. ulcerans*, homogenization was achieved by rigorously vortexing with 3-mm glass beads (Sigma-Aldrich, Saint-Quentin-Fallavier, France) followed by four bypasses through a 25-G needle to disperse the remaining clumped bacilli. Homogenized suspensions were calibrated using spectrophotometry to a 1 Mc Farland (McF) unit, equivalent to a 10^7^ colony-forming units (CFU)/mL inoculum for non-clumping mycobacteria and a 10^6^ CFU/mL inoculum for clumping mycobacteria.

### Culture

Middlebrook 7H10 medium (Becton–Dickinson, Le Pont-de-Claix, France) was supplemented with sodium chloride (NaCl) (Sigma-Aldrich, Saint-Quentin-Fallavier, France) in 1 % increasing salt concentrations from 0 (weight/volume) to 10 %. A 100 µL-volume containing 10^4^ CFUs was inoculated in triplicate on 90-mm sterile plates containing Middlebrook 7H10 medium (control) or Middlebrook 7H10-NaCl-supplemented media. Plates inoculated with *M. tuberculosis*, “*M. canettii”*, *M. bovis* BCG, *M. avium*, *M. hominissuis*, *M. intracellulare* and *M. chimaera* were incubated at 37 °C in a 5 %-CO_2_ atmosphere. Plates inoculated with *M. abscessus*, *M. chelonae*, *M. boletti*, *M. massiliense*, *M. fortuitum*, *M. conceptionense* and *M. lentiflavum* were incubated at 35 °C in ambient air as previously described [9]. Plates inoculated with *M. ulcerans* and *M. marinum* were incubated at 30 °C in ambient air. Plates were checked by visual inspection weekly for colonies for 4 weeks. Colonies were counted regardless of their size and counting was considered interpretable when >10^3^ colonies were observable on the control (0 %-NaCl) Middlebrook 7H10 medium plates. A strain was considered salt tolerant when more than 50 colonies developed on the 7H10-NaCl supplemented media. Image J program [10] was used to measure the average size of colonies after 50 colonies were randomly chosen from each plate. The morphology of colonies was observed by the naked-eye. Ziehl-Neelsen staining was conducted to confirm the identity of the colonies.

### Genome analyses

The whole genome and proteome of 15 mycobacterial species under investigation were downloaded from Genbank (Table 1). The proteins were clustered into orthologous groups using orthoMCL [11] with a conservative parameter value of 60 % sequence identity. Homologous sequences were selected using the all-against-all BlastP algorithm [12] with an E value of <10^−5^. Clustering of the orthologous sequences was then analyzed using the Markov Cluster algorithm [13]. Determination of the different unique core genomes was based on the homology clusters found by orthoMCL. The resulting orthologous groups were used to construct a whole-genome tree using the Neighbor-Net algorithm based on a gene content matrix using splitree [14]. The similarity between two species was defined as the number of genes in common divided by the total number of genes of the two species [15].

**Table 1:** Genome properties and pan**-**genome analysis

Genome	GenBank accession N°	GC %	N° of CDS	N° of CDS in groups	Unique core genes	Core genome	Core genes	Pan genome	Pan genes
*Group 1 (Sensitive)*	290					1563	37,132	6043	70,580
*M. tuberculosis* H37Rv	NC_00962	65.6	3906	3863	45				
*M. tuberculosis* str.Beijing/NITR203	NC_021054	66	4110	1969	141				
*M. tuberculosis* str. Erdman = ATCC 35801	NC_20559	65.6	4245	3960	285				
*M. canettii* CIPT 140010059	NC_015848	66	3861	3796	81				
*M. bovis* BCG str. Pasteur 1173P2	NC_008769	66	3949	3884	67				
*M. marinum* M	NC_010612	66	5423	5092	349				
*M. ulcerans* Agy99	NC_008611	65	4160	4070	310				
*Group 2 (Intermediate)*	0								
*M. avium* 104	NC_00859	69	5120	4817	311				
*M. intracellulare* ATCC 13950	NC_016946	68	5144	4777	380				
*M. massiliense* str. GO06	NC_018150	64	4558	4446	112				
*Group 3 (resistant)*	0								
*M. abscessus* T	NC_010397	64	4920	4701	223				
*M. abscessus* subsp. *bolletii* BD	AHAS01000000	64	4923	4657	268				
*M. abscessus* subsp. *bolletii* CCUG 48898	AKVF010000000	64	5511	4867	656				
*M. avium* subsp. *hominissuis* A5	AUZQ01000000	69	4509	4401	113				
*M. fortuitum* subsp. *fortuitum* DSM 46621	ALQB01000000	66	6241	5373	985				

## Results

### Salt tolerance of mycobacteria

In the MTC, two *M. tuberculosis* clinical isolates grew up to 1 % only whereas 10 other *M. tuberculosis* clinical strains including two Beijing strains and *M. tuberculosis* H37Rv reference strain grew up to 3 %. *M. bovis* BCG grew up to 2 % and three “*M. canettii*” isolates grew up to 3 %. In the *M. avium* complex, *M. chimaera* grew up to 4 %, *M. avium* and *M. intracellulare* up to 5 % and two *M. hominissuis* isolates up to 7 %. In the *M. chelonae*-*abscessus* complex, *M. chelonae* grew up to 3 %, *M. massiliense* up to 4 %, *M. abscessus* and *M. bolettii* up to 7 %. Then, *M. marinum* and two *M. ulcerans* reference strains grew up to 3 %. Further, *M. lentiflavum* grew up to 1 %, *M. conceptionense* grew up to 7 % and *M. fortuitum* grew up to 8 % (Tables 2, 3). Based on these results, we defined three groups of mycobacteria: Group 1 includes “salt-sensitive” species (growth up to ≤3 % salt); Group 2 includes the “salt-intermediate” species (growth up between to 4 and 6 % salt), while Group 3 includes “salt-resistant” species (growth up to >6 % salt).

**Table 2:**
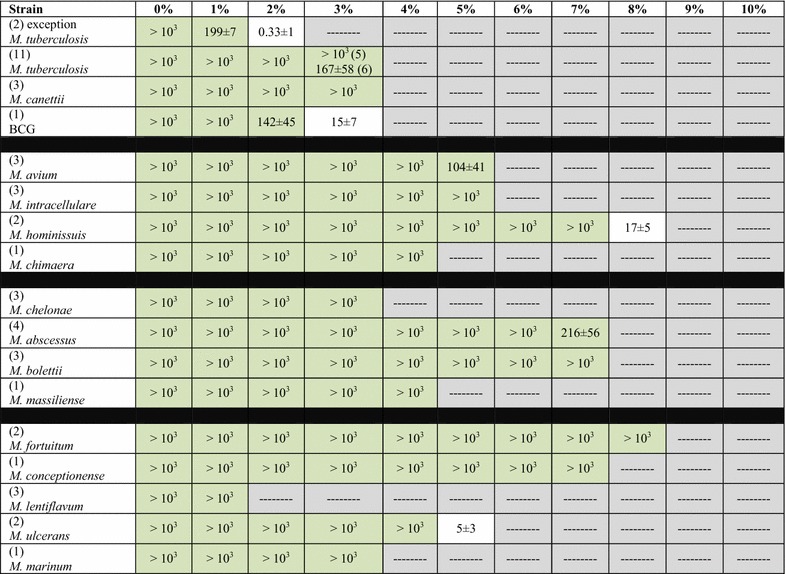
Number of colonies detected after four-week incubation on Middlebrook 7H10 medium incorporating increasing salt concentrations

green, growth; white, negative (<50 colonies)

**Table 3:**
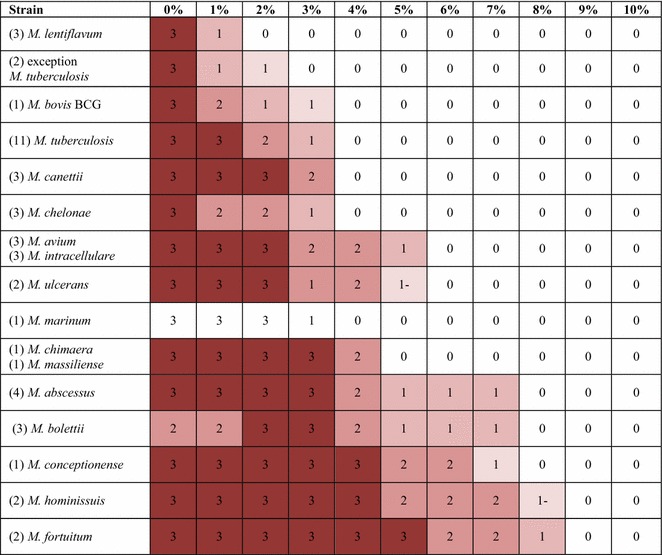
Representation of the colonies size on the different medium

3, biggest, 2, 1, smallest; 1-, sometimes an absence of colonies

### Colony size and morphology

**Fig. 1:**
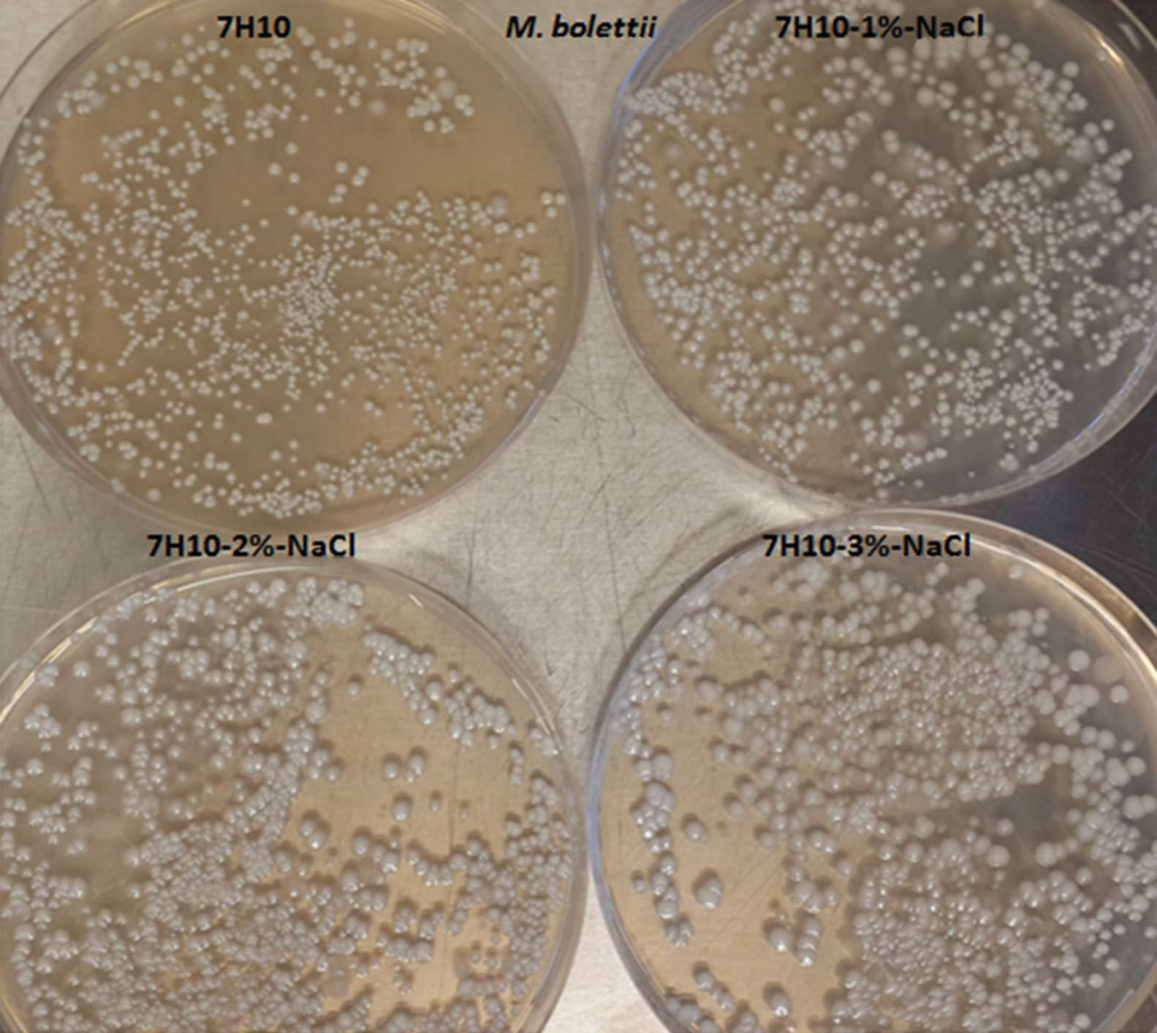
*M. bolettii* colonies on 7H10, 7H10-1 %-NaCl, 7H10-2 %-NaCl and 7H10-3 %-NaCl

Except for *M. bolettii*, we observed that the size of colonies significantly decreased as salinity increased (Table 3). As for *M. bolettii*, the size of colonies increased from 1.16 ± 0.4 mm in the Middlebrook 7H10 control medium up to 2.95 ± 0.9 mm in 3 % NaCl Middlebrook 7H10 medium (P < 0.05, student’s *t* test), then decreased down to 0.4 ± 0.2 mm in 7 % NaCl Middlebrook 7H10 medium (P < 0.05, student’s *t* test) (Fig. 1; Table 4).

**Table 4:**
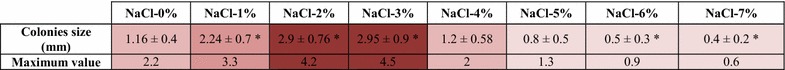
Size of colonies (mean ± standard deviation and maximum value) for *M. bolletii* grown on Middlebrook 7H10 medium enriched in increasing concentration of NaCl

Denotes P < 0.05 when compared to NaCl-0 %

### Genome analysis

**Fig. 2:**
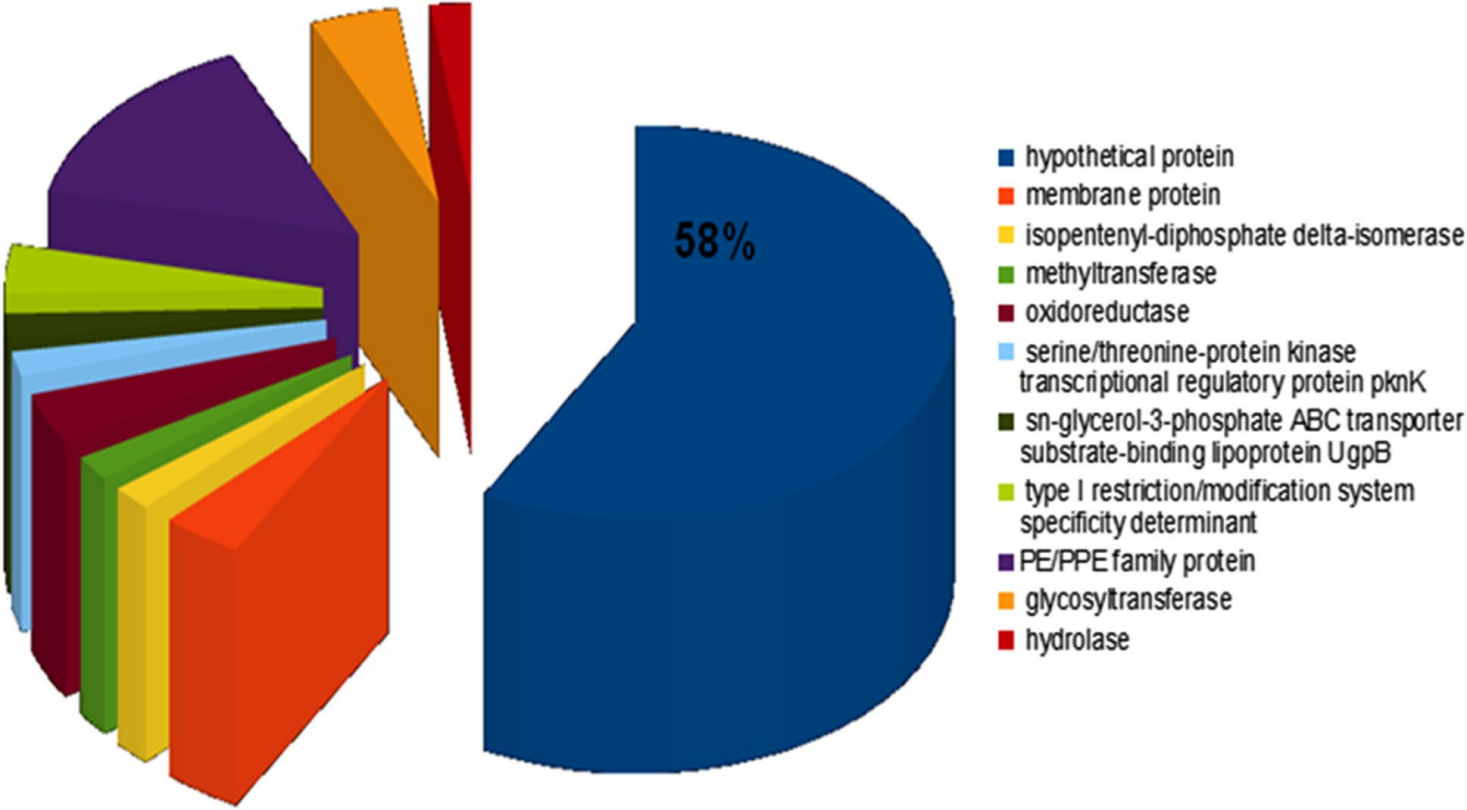
Pie-chart representing the cluster of orthologous genes (COG) classification of 290 core genes in Group 1 mycobacteria

Of the 15 analyzed *Mycobacterium* species in this study, we identified a total of 70,580 protein-coding sequences, varying from 3861 protein-coding sequences in “*M. canettii*” to 6241 in *M. fortuitum* (Table 1). The core-genome contains 37,132 protein sequences accounting for 52 % of the pan-genome. These 70,580 proteins were characterized into 6043 orthologous protein groups, including 1563 core-genome groups and 91 strain-specific groups. Furthermore, using orthoMCL clustering, no unique genes (gene shared only by the genome of species belonging to the same group) were found in Group 2 and Group 3. However, 290 genes were unique to species of the Group 1, with 58 % of these genes being annotated as hypothetical proteins while the other 42 % were Pro-Glu (PE) and Pro–Pro-Glu (PPE) family proteins (14.8 %), glycosyltransferase (4.5 %), type I restriction/modification system specificity determinant (4.1 %), oxidoreductase (4.1 %), membrane protein (4.1 %), isoprentenyl-diphosphate delta-isomerase (2.08 %), methyltransferase (2.08 %), serine/threonine-protein kinase transcriptional regulatory protein pknK (2.08 %), sn-glycerol-3-phosphate ABC transporter substrate-bonding Ipo protein Ugp B (2.08 %), hydrolase (2.08 %) (Table 5; Fig. 2). Studies reported that general stress tolerance proteins such the Glucose starvation inducible Protein B (GsiB) and a putative enoyl-CoA hydratase (EchM) [16], Na+/H+ efflux pumps proteins [17], porin and genes implicated in porin regulation [18] are involved in tolerance of toxic compound including salt. However only three proteins were found to harbor the GsiB domains with low identity <30 % and annotated as two extracellular solute-binding protein (*M. fortuitum* and *M. avium*) and one peptide ABC transporter substrate-binding protein (*M. hominissuis*). Also, from one to four Na+/H+ antiported and transporter proteins were found in *M. abscessus, M. bolleti*, *M. massiliense*, *M. avium*, *M. intracellulare*, *M. fortuitum* and *M hominissuis*. From one to three porin precursor and one aquaporin proteins were found in *M. abscessus, M. bolleti*, *M. massiliense*, *M. fortuitum* and *M. massiliense.*

**Table 5:** Clusters of orthologous genes (COG) classification of 290 core genes specifically found in Group 1 mycobacteria

Gene annotation	Number of genes	Genes percentage in Pan genome
Hypothetical protein	168	57,9,310,344,828
PE-PPE family protein	43	14,8,275,862,069
Glycosyltransferase	13	4,4,827,586,207
Membrane protein	12	4,1,379,310,345
Oxidoreductase	12	4,1,379,310,345
Type I restriction/modification system specificity determinant	12	4,1,379,310,345
Isopentenyl-diphosphate delta-isomerase	6	2,0,689,655,172
Serine /Threonine-protein kinase transcriptional regulatory protein pknK	6	2,0689655172
Sn-glycerol-3-phosphate ABC transporter substrate-binding lipoprotein UgpB	6	2,0,689,655,172
Methyltransferase	6	2,0,689,655,172
Hydrolase	6	2,0,689,655,172

There was no unique gene for Group 2 and Group 3 mycobacteria

## Discussion

We observed a previously unrated wide range of salt tolerance for mycobacteria, from 1 % (*M. lentiflavum*) to 8 % (*M. fortuitum*) among mycobacteria of veterinary and medical interests comprising of obligate and opportunistic pathogens. Data here reported were authenticated by being reproduced in triplicate. Furthermore, data here obtained for *M. abscessus* and *M. chelonae* agree with the previously reported 50 g/L salt tolerance for the same species using a comparable methodology [9]. Likewise, the 5 % limit here found for *M. intracellulare*, has been previously reported using a slightly different methodology [9]. The data here reported for *M. tuberculosis* also agree with those previously reported for *M. tuberculosis* and *Mycobacterium bovis* using the BioLog technique [19]. In a later study*, M. tuberculosis* H37Rv as well as a *M. tuberculosis* Beijing strain were shown to be highly susceptible to salt with metabolic activity exponentially dropping as salt concentration increased from 0 to 3 % [19]. Accordingly, we observed that *M. tuberculosis* growth was inhibited by salinity rate >3 %.

In this study, we observed that *M. tuberculosis* complex members tolerate up to 3 %, most *M. avium* complex members between 4 and 5 % and *M. abscessus* and *M. bolettii* up to 7 %. Furthermore, variations were observed within these phylogenetic complexes, as previously reported for *M. tuberculosis* and *M. bovis* in the *M. tuberculosis* complex [19]. In the *M. avium* complex, *M. chimaera* exhibits a salt tolerance limited to 3 %, which is much lower than that of *M. avium* subsp. *homnissuis* which is limited to 7 %-NaCl. The same observation holds true for the *M. chelonae*-*abscessus* complex where *M. chelonae* exhibits a 3 % tolerance whereas *M. massiliense* grew up to 4 % and *M. bolettii* up to 7 %. It is worth noting that these two species yielded quite different colony morphology, which was smooth for *M. bolettii* and rough for *M. abscessus*. Such notable differences in salt-tolerance could be further incorporated in the easy identification of colonies in clinical microbiology, as previously reported [9].

**Fig. 3:**
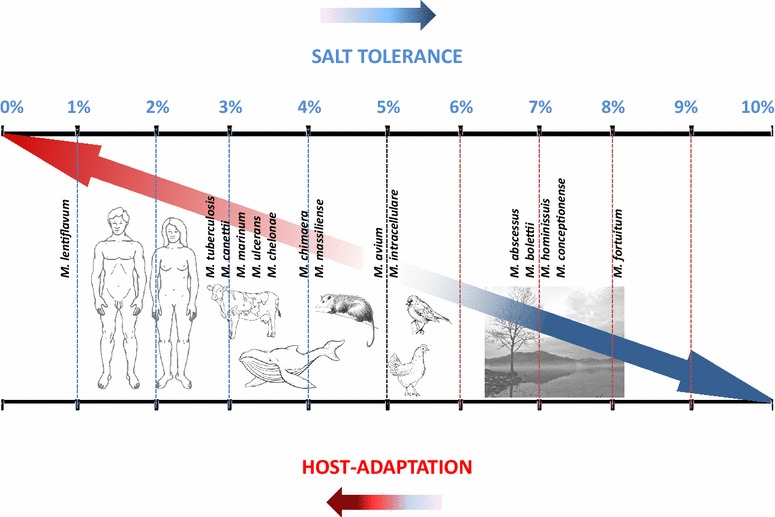
Inverse correlation between salt tolerance and host adaptation in mycobacteria

Rather than observing a correlation with the phylogenetic position, we observed a correlation with the genome content and encoding capacity. Indeed, we found that Group 1 salt-susceptible mycobacteria contained a 290-gene core that is absent in salt-tolerant groups. It is worth noting that more than one-third of these genes encode for PE/PPE family proteins. PE/PPEs are functionally associated with type VII or ESX secretion systems and could act as virulence factors helping the bacteria to establish a successful infection inside the host [20–22]. We therefore observed an inverse correlation between salt tolerance and host adaptation (Fig. 3). In particular, Group 1 contained only species associated with the infection of terrestrial mammal organisms such as *M. tuberculosis*, *M. canettii*, *M. bovis*, *M. lentiflavum* and *M. chelonae*; and the infection of marine mammal organisms, notably whales such as *M. marinum* (formally *Mycobacterium balnei*) [23]. Even whales maintain low serum sodium concentration (152 mEq/L) similar to the salt concentration (135–145 mEg/L) measured in terrestrial mammals [24]. General stress tolerance proteins [16], Na+/H+ efflux pumps [17], porins and genes implicated in porin regulation [18] are of utmost importance for bacteria in order to adapt to environmental changes such as the presence of toxic compounds including salt. Strangely, only three proteins harboring the GsiB domain and a putative enoyl-CoA hydratase (EchM) previously reported to be responsible for salt tolerance [16] were found with low identity <30 % in *M. fortuitum*, *M. avium* and *M. hominissuis*, which tolerate salt. Na+/H+ efflux pumps proteins and porins were found only in strains which are intermediate or resistant to salt.

## Conclusions

In conclusion, we propose that salinity is one of the multiple factors which determine the ecological niches of mycobacteria, with tolerance to salt being roughly inversely correlated with host adaptation.

## Abbreviations

MTC: *Mycobacterium tuberculosis* complex
PE: proline- glutamic acid
PPE: proline-proline-glutamic acid
BCG: bacillus calmette guerin
CIP: collection institut pasteur
MAC: *Mycobacterium avium* complex
ATCC: American type culture collection
PCR: polymetre chain reaction
PBS: phosphate buffered saline
CFU: colony-forming unit
